# Hierarchical automated machine learning (AutoML) for advanced unconventional reservoir characterization

**DOI:** 10.1038/s41598-023-40904-0

**Published:** 2023-08-24

**Authors:** Yousef Mubarak, Ardiansyah Koeshidayatullah

**Affiliations:** 1 Department of Geosciences, College of Petroleum Engineering and Geosciences, King Fahd University of Petroleum and Minerals, Dhahran, Saudi Arabia; 2 Saudi Aramco, Dhahran, 31311 Saudi Arabia; 3 Center for Integrative Petroleum Research, College of Petroleum Engineering and Geosciences, King Fahd University of Petroleum and Minerals, Dhahran, Saudi Arabia

**Keywords:** Geology, Energy science and technology

## Abstract

Recent advances in machine learning (ML) have transformed the landscape of energy exploration, including hydrocarbon, CO_2_ storage, and hydrogen. However, building competent ML models for reservoir characterization necessitates specific in-depth knowledge in order to fine-tune the models and achieve the best predictions, limiting the accessibility of machine learning in geosciences. To mitigate this issue, we implemented the recently emerged automated machine learning (AutoML) approach to perform an algorithm search for conducting an unconventional reservoir characterization with a more optimized and accessible workflow than traditional ML approaches. In this study, over 1000 wells from Alberta’s Athabasca Oil Sands were analyzed to predict various key reservoir properties such as lithofacies, porosity, volume of shale, and bitumen mass percentage. Our proposed workflow consists of two stages of AutoML predictions, including (1) the first stage focuses on predicting the volume of shale and porosity by using conventional well log data, and (2) the second stage combines the predicted outputs with well log data to predict the lithofacies and bitumen percentage. The findings show that out of the ten different models tested for predicting the porosity (78% in accuracy), the volume of shale (80.5%), bitumen percentage (67.3%), and lithofacies classification (98%), distributed random forest, and gradient boosting machine emerged as the best models. When compared to the manually fine-tuned conventional machine learning algorithms, the AutoML-based algorithms provide a notable improvement on reservoir property predictions, with higher weighted average f1-scores of up to 15–20% in the classification problem and 5–10% in the adjusted-R^2^ score for the regression problems in the blind test dataset, and it is achieved only after ~ 400 s of training and testing processes. In addition, from the feature ranking extraction technique, there is a good agreement with domain experts regarding the most significant input parameters in each prediction. Therefore, it is evidence that the AutoML workflow has proven powerful in performing advanced petrophysical analysis and reservoir characterization with minimal time and human intervention, allowing more accessibility to domain experts while maintaining the model’s explainability. Integration of AutoML and subject matter experts could advance artificial intelligence technology implementation in optimizing data-driven energy geosciences.

## Introduction

Subsurface well log data can provide critical information on the spatial and temporal variability of depositional lithofacies and petrophysical properties of reservoir zones, allowing for a more complete reservoir evaluation^1–3^. In addition, well log data is typically more abundantly available in most wells than other subsurface data, such as cores. Despite its efficiency, well logging has some limitations when it comes to the level of uncertainty in heterogenous depositional settings and the needs of experienced petrophysicists to perform the data processing and interpretation^4,5^. In hydrocarbon exploration, petrophysical analysis, such as lithofacies classification and porosity prediction, is one of the most active areas where machine learning can be applied^6,7^. This is primarily because petrophysical data is well structured and well defined in terms of physical models. As a result, many sophisticated machine learning algorithms can be applied to petrophysical data^7^. This is further empowered by the emergence of artificial intelligence technology and the availability of massive volumes of subsurface datasets have paved the way for advanced machine learning algorithms. Following the seminal work by Wong^8^ which provided the one of the earliest successful attempts in applying artificial neural networks to predict porosity, numerous attempts have been introduced in using various supervised and unsupervised machine learning models to predict different petrophysical properties. Al-Anazi and Gates^9^ used support vector regression for predicting porosity in heterogenous reservoir. Furthermore, Chen et al.^10^ has implemented a deep learning algorithm to predict porosity. The approach could reduce errors when limited data is available and different log depths are present. Recently, a study by Yang^11^ utilized state-of-the-art deep learning transformer model to predict porosity and achieved high accuracy. Several works have also extended the application of machine learning to conduct permeability predictions in both siliciclastic and carbonate reservoirs^12,13^.

One of the main challenges in well log interpretation is to determine the lithofacies from various log responses. While statistical approaches have been implemented to aid the classification, it is often inaccurate and does not provide a good agreement with the core description^14^. Qi and Carr^15^ provided one of the initial attempts to use unsupervised clustering to classify lithofacies based on well-log responses (e.g., density, gamma ray). Hall^16^ published a seminal paper on using machine learning to predict facies using a type of supervised machine learning known as support vector machine. This study used a dataset of nine wells that included data from seven well logs (e.g., GR, Neutron, and Density) to predict nine different facies. The model’s output showed a moderate level of accuracy, but it still requires more work to improve. Nonetheless, this work has demonstrated a systematic and easy-to-follow workflow to predict facies using machine learning models. Since then, most workflows have included advanced machine learning algorithms to automatically interpret depositional facies from well logs, including carbonate reservoirs^17,18^. Bestagini et al.^19^ proposed a supervised machine learning technique using well logs to predict various facies. In this case, the proposed model divides the training sets observations/features into distinct subsets. Then only a few features from each subset are used to train the decision tree using cost function concepts. This technique has a high level of accuracy and has the potential to improve prediction performance by adding more geological constraints^19^. In contrast to previous studies that focused solely on one method/algorithm, Ippolito et al.^20^ proposed a hybrid machine learning model to predict facies. To overcome bias issues, this study combines heterogeneous features of supervised machine learning and unsupervised machine learning. Such algorithms are now increasingly implemented to predict subsurface reservoir properties^16,18,21^. Jaikla et al.^22^ proposed a *FaciesNet* algorithm for lithofacies prediction based on deep recurrent neural networks. This study shows a notable improvement in the overall performance when utilizing deep learning for facies prediction.

Despite these advances, it is worth noting that developing and training machine learning algorithms takes time and requires expertise outside of geosciences to conduct the necessary data pre-conditioning to run such machine learning models^16,23–25^. Furthermore, time-consuming processes such as data preparation and processing, selecting appropriate parameters, and fine-tuning the model are frequently required to test and compare different classification algorithms. Therefore, the implementation of machine learning is often limited to specific fields with high levels of ambiguity and non-accessible for non-machine learning experts. To overcome this issue, several works have proposed different workflows to automate model generation, tuning and evaluation processes, or to create an automated machine learning (AutoML) approach^26,27^. In such a case, AutoML focuses on hyperparameter optimization and model optimization by using Bayesian optimization, genetic algorithms, or reinforcement learning^25^.

While recent studies have shown the prospect of AutoML in optimizing the overall machine learning pipeline and achieving high accuracy predictions^28,29^, the application of AutoML in geosciences remains limited. To date, there is only a few works attempted to use AutoML for subsurface petrophysical analysis^30^. As a result, the primary goal of this research is to investigate and assess the feasibility of AutoML for generating various petrophysical analyses and reservoir property predictions. The motivation for using AutoML is to create a model that can predict lithology and other reservoir properties with minimal human intervention and is transferable across disciplines due to the fact that it requires non-ML experts to test the model. In this study, we focused on the subsurface well log dataset from the Cretaceous Athabasca oil sands to conduct advanced unconventional reservoir characterization with AutoML. The proposed hierarchical AutoML workflow is divided into two main stages: (1) the first stage is to predict volume of shale, porosity, and bitumen percentage from available well log data (e.g., gamma ray, density, and neutron) and (2) the second stage is focused on lithofacies classification by using the original well log data and predicted outputs from the first stage. Finally, the study will compare and contrast the performance and efficiency of traditional and automated machine learning models.

## Dataset and methodology

### Athabasca oil sands

**Figure 1:**
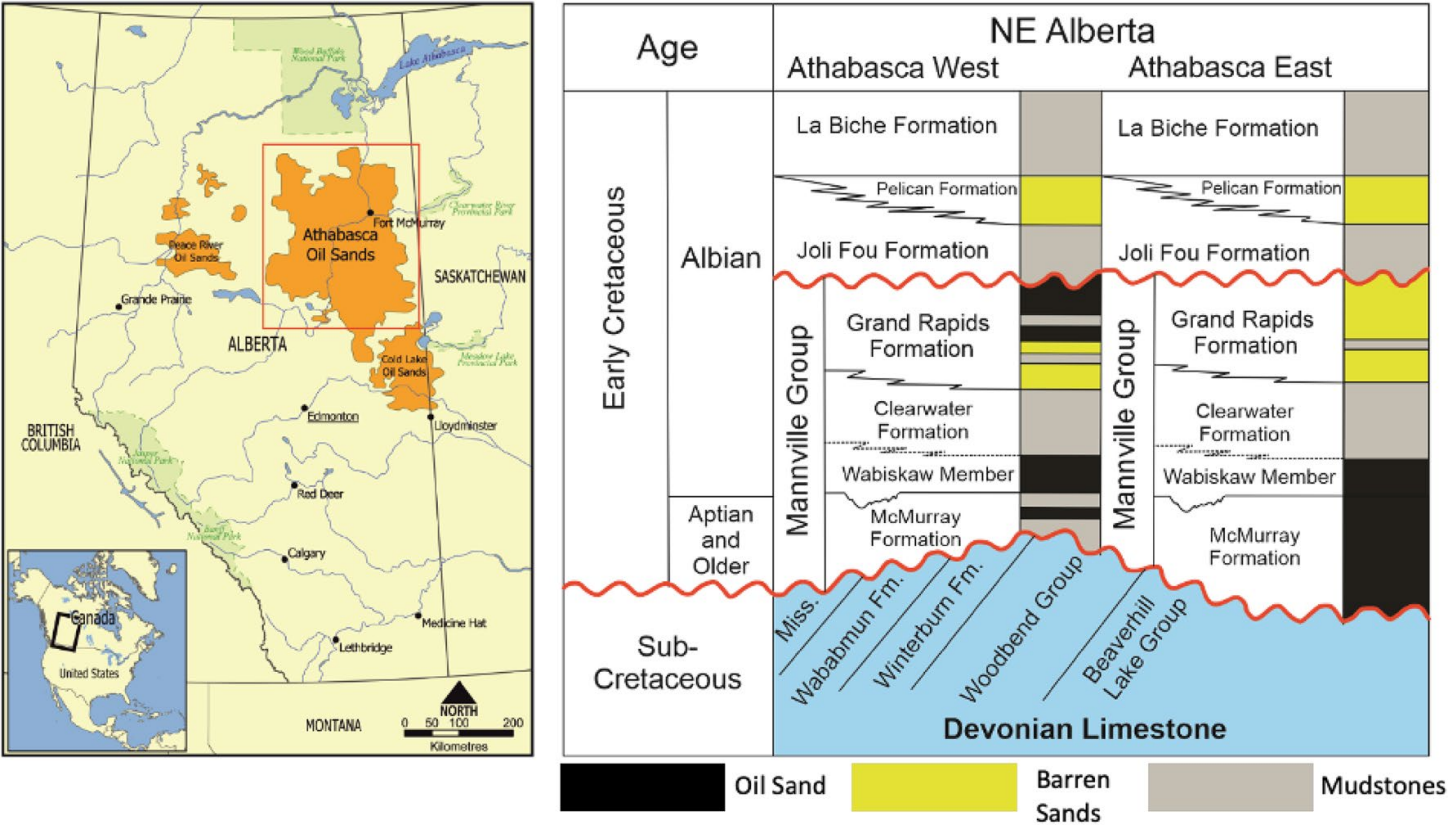
Location of four major oil sand deposits in Alberta, Canada^32^.

The study area is located in the Athabasca oil sands in Alberta, Canada, which is considered one of the world’s largest bitumen deposits^31^. The majority of these bitumen resources were discovered in four major deposits: Athabasca, Cold Lake, Wabasca, and Peace River^32^ (Fig. 1). With estimated resources of around 1 trillion barrels of bitumen, the Athabasca is considered the world’s largest bitumen deposit^33,34^. These deposits are part of the Western Canada sedimentary basin, which is bounded on the west by the Rocky Mountains and on the east by the Canadian shield and is divided into two sections: the Williston intracratonic basin in the southwest and the Alberta foreland basin (Fig. 1). The basin was formed during the Paleozoic rifting period, which was followed by the development of a passive margin due to thermal subsidence^35^. Devonian-aged mixed succession of carbonate, evaporites, and shales deposited along the passive margin are the oldest preserved sediments in the Athabasca oil sand deposits. As a result, several studies suggest that these Devonian shales may be a source rock for the Athabasca Oil Sand^36^. This was followed by a period of siliciclastic deposition from the Late Paleozoic to the Late Jurassic, which could have resulted in the formation of Jurassic source rock^35^. The development of the Rocky Mountains fold and thrust belt, which controlled the deposition of the foreland basin megasequence, resulted in a significant shift in sediment provenance during the Late Jurassic.

This megasequence was dominated by siliciclastic deposition during the Early Cretaceous, and it includes the Lower Cretaceous Mannville group reservoirs, the primary reservoir interval in the Athabasca oil sands^35^. The McMurray formation, which unconformably overlies the Devonian carbonate, is the first Mannville group sedimentary unit found in Alberta, followed by the Wabiskaw member of the Clearwater Formation, which sits unconformably on the McMurray Formation^33^ (Fig. 1). The primary reservoirs in the Athabasca oil are the McMurray–Wabiskaw clastic deposits, which are then capped by the Clearwater Formation shales as the ultimate regional seal^37^. In general, the McMurray–Wabiskaw interval is composed primarily of a deepening-upward complex system of sediments controlled by a sub-Cretaceous unconformity configuration^37^. These deposits are primarily composed of four facies associations: fluvial, tidal flat, tidal bar complex, and tidal bar cap^38^. The McMurray and Wabiskaw reservoirs have a thickness of up to 40 m and a porosity of up to 30%^39^. The majority of Athabasca oil sand is hosted in the Lower Cretaceous McMurray–Wabiskaw interval, from which the majority of bitumen resources can be recovered using thermal in-situ and surface mining methods^40^.

### Well log data

**Figure 2:**
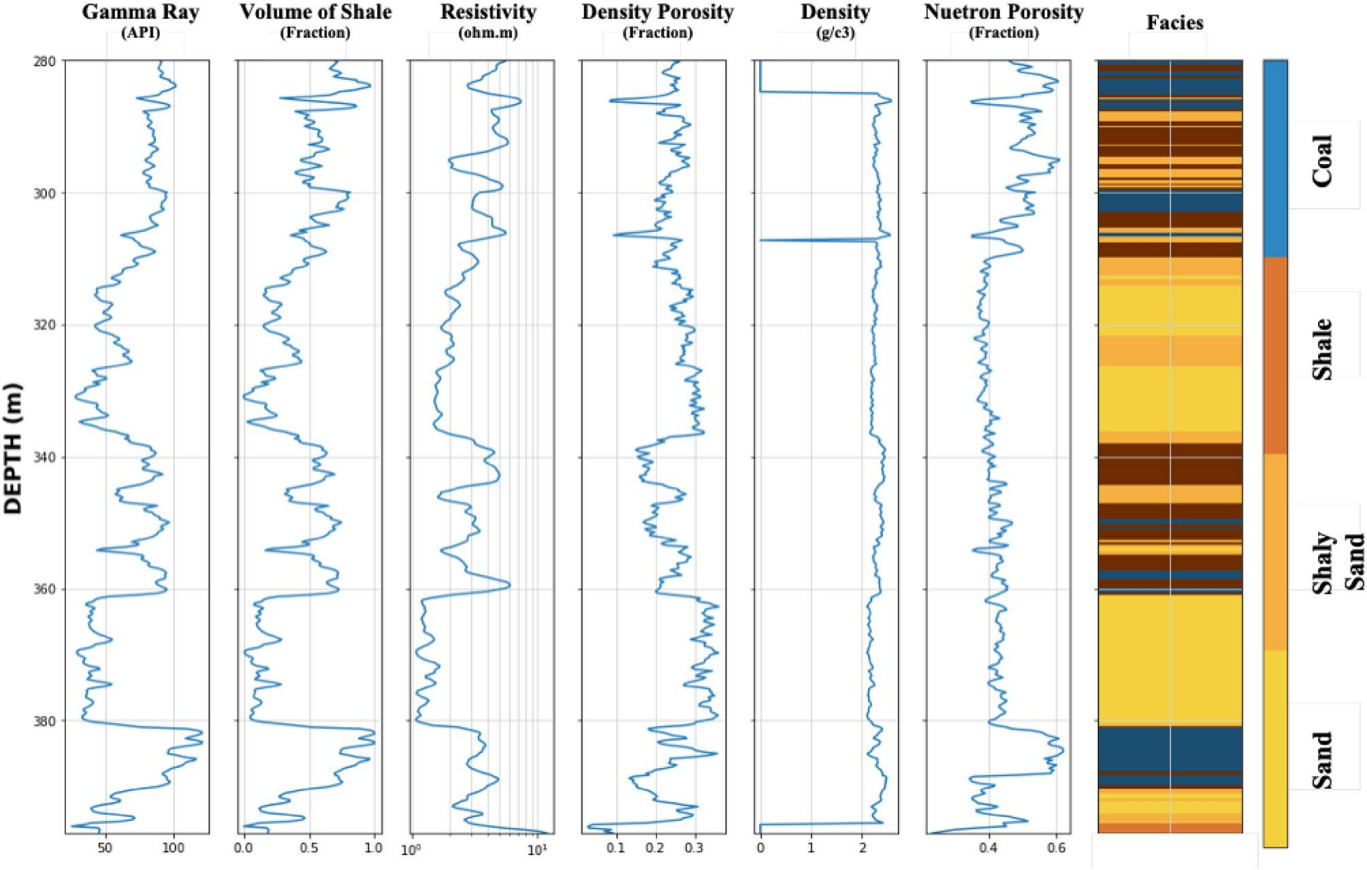
Examples of the available well data and lithofacies interpretation in the datasets.

This study utilized a publicly available dataset of 2173 wells provided by the Alberta geological survey as part of a regional study conducted in 1985. The primary goal of acquiring this dataset was to map the Lower Cretaceous McMurray Formation and the overlying Wabiskaw member of the Clearwater Formation in Alberta, Canada’s Athabasca Oil Sand area. The following data are available for petrophysical and other measurements: lithology log (LITH), bitumen mass percentage (W_Tar), water saturation (Sw), shale volume (VSH), porosity (PHI), and water resistivity (Rw). A suite of well logs with variable coverage, such as Gamma ray (GR), Resistivity (ILD), Caliper (CALI), Density (RHOB), Neutron (NPHI), and Porosity derived from density (DPHI), is also available (Fig. 2). There are four distinct lithologies identified using 750 wells and core data analysis (Sand, Shaly Sand, Shale, and Coal; Fig. 2). According to the attached report from the Alberta Geological Survey in 1994, the interpreted lithology log was then populated using various petrophysical equations, primarily volume of shale and porosity calculated using density and neutron logs.

### Exploratory data analysis

**Figure 3:**
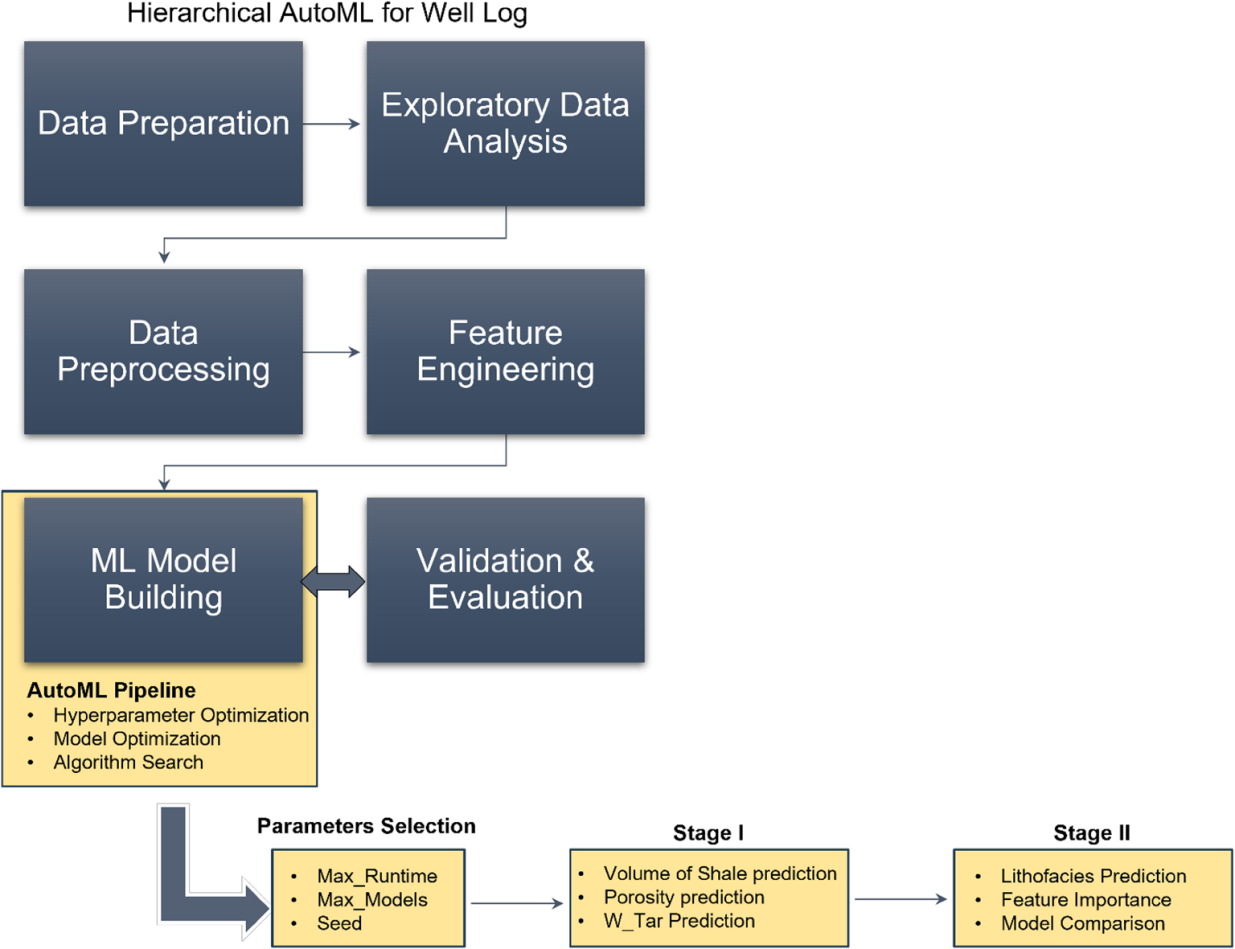
Conventional machine learning and AutoML workflow.

**Figure 4:**
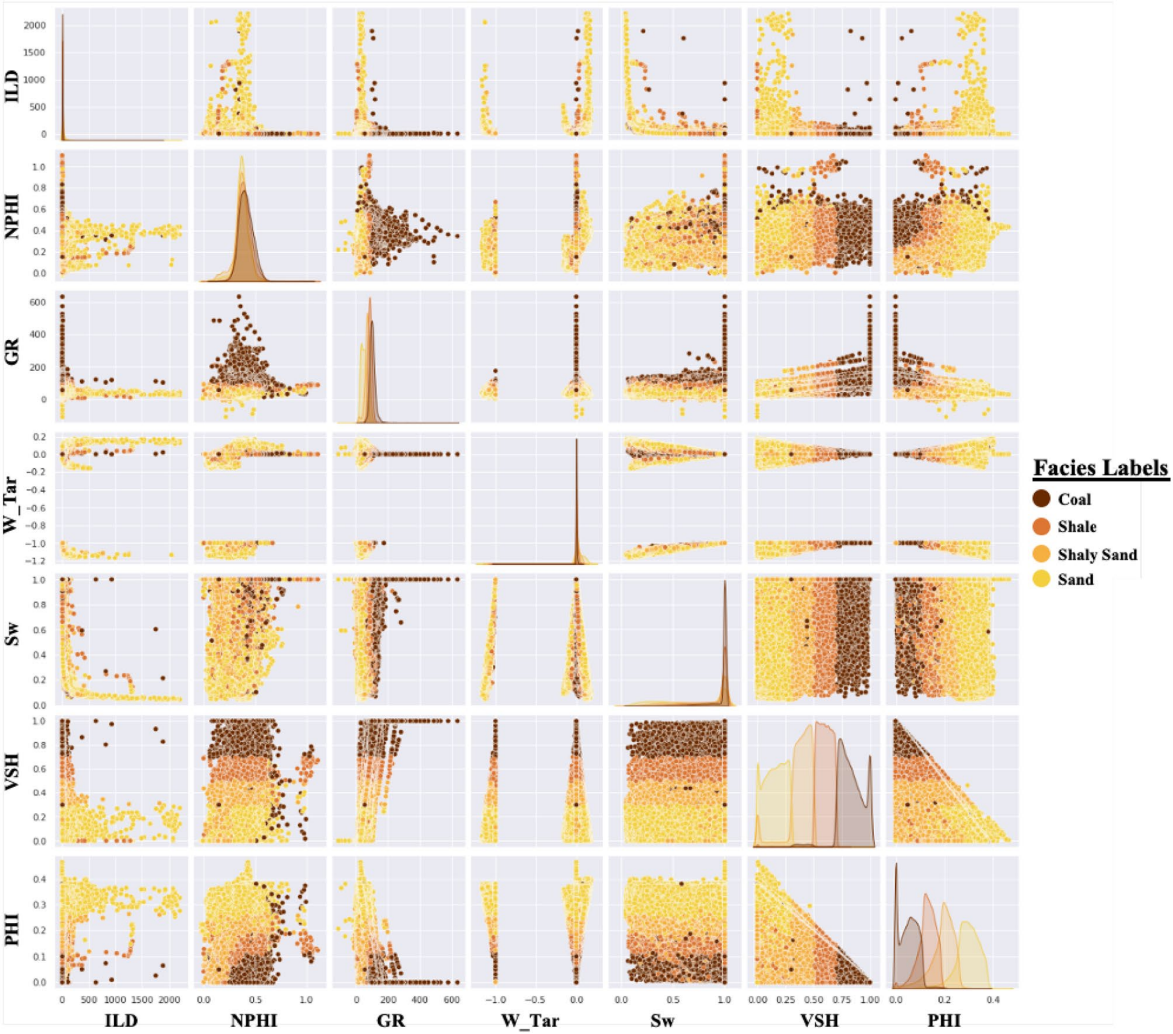
Cross plots between different parameters available in the dataset. It is evident that different lithologies show different log responses and variable results in the laboratory measurements.

In this study, we followed a standard exploratory petrophysical data analysis workflow to preprocess the data and unravel any statistical patterns/trends (Fig. 3). Python programming language and built-in libraries (e.g., pandas, scikit-learn) were used to process and analyze the available data. Because this study involves a large number of well data, data cleaning was performed by sorting, rescaling, grouping, and reformatting to ensure the data is uniform and ready for machine learning analysis (Fig. 3). In addition, data preparation required analyzing the outlier values/trends observed in the well log values using log normalization across different wells, removing outliers, and scaling for consistency. To avoid miscalculation and error during machine learning training and prediction, all missing values were removed from the dataset. The exploratory data analysis was carried out using various visualization techniques such as cross-plots and histograms. This step is critical for identifying patterns and analyzing anomalous values using descriptive statistics. It is also useful for determining the significance of certain features in order to aid in the prediction of logs based on the identified relationship which can be recognized in Fig. 4.

### Machine learning

#### Supervised machine learning

Several supervised machine learning models were evaluated and compared as a baseline model with the AutoML model in this study. Both logistic regression and gradient boosting machine classifier were used for the discrete task (facies prediction); while linear regression and gradient boosting machine regressor were used to predict continuous data. For example, gradient boosting machine is utilized for VSH and W_Tar, while random forest regressor is utilized for PHI. The total dataset was divided into 80% training and 20% blind test for all learning techniques. The training dataset was further divided into 80% for training and 20% for validation. The data for training and validation was completely separated from the test set in order to get independent results. Then the following set of logs were used as training features: GR, DPHI, NPHI and ILD to predict the lithology log. The same input logs were also used to predict the VSH and W_Tar.

Breiman^41^ first introduced the random forest (RF) algorithm as an ensemble supervised machine learning algorithm that relies on decision trees. In each tree, RF combines bagging and different bootstrapping processes, adding an extra layer of randomness to the model. Furthermore, while the RF algorithm is inspired by the decision tree algorithm, it introduces randomness in separating each node and selecting the best predictors in that node^42^. Overall, when compared to Decision Tree, RF reduces overfitting and its performance is robust to outliers in the dataset^42,43^. Gradient boosting machine (GBM) is a concept that was developed to iteratively improve the performance of weak learners and create an efficient learner^44,45^. In general, GBM consists of three key components: (1) loss function optimization; (2) a weak learner, which is typically a decision tree, to make predictions; and (3) an additive model to add weak learners to minimize loss function. The main advantage of GBM is its ability to work with large and complex datasets, as well as its robustness to bias and outliers in the dataset. However, GBM, like RF, can be costly to train and tune. Furthermore, GBM is known to suffer from model overfitting to training datasets, so regularization methods (L1 and L2), as implemented in the extreme gradient boosting algorithm (XGB), are required to mitigate this issue.

### Automated machine learning (AutoML) implementation

Recent advances in artificial intelligence technologies enable the development and implementation of automated machine learning (AutoML), which automates the architectural design, selection, and parameterization of machine learning models^26,46^. In this study, we chose the open-source, distributed machine learning platform built to scale to large datasets, H_2_O tool for AutoML because of its scalability, user-friendliness, versatility, and extensive libraries to explore the models^47^. In this case, AutoML employs a combination of random grid search and stacked ensembles, as diverse models improve the accuracy of the ensemble method. To make the tool accessible to non-experts, in this study, only a few parameters are required to train the model within the H_2_O tool. These parameters serve as constrains for the AutoML process, so as soon as any of them is met, the AutoML process will stop:

▪ *Max_runtime_secs*: This constrain is to specify the amount of time the AutoML process will run to train various models (ex. Generalized linear model (GLM), Gradient boosting (GBM) and distributed random forest (RDF)). Followed by finetuning associated hyperparameters and evaluating best models based on certain metrics (ex. Root mean square). This is solely based on predefined parameters until the runtime is achieved.

▪ *Max_models*: this is to specify the number of models to be included in the AutoML process. This is an exception to Stacked ensemble models that basically tries to combine the different models and get best results.

▪ *Seed*: This option specifies the random number generator (RNG) seed for algorithms that are dependent on randomization.

In this work, the following conditioned were applied while running H_2_O AutoML learning modelling including the training and validation process: max_models = 10, max_runtime_sec = 400, seed = 1234. In addition, we excluded the stacked ensemble model generated by the H_2_O model to allow a fair comparison with other conventional ML models.

#### Evaluation metrics

The models were evaluated by using various evaluation metrics such as adjusted coefficient determination (adjusted R^2^; Eq. 1), root mean squared error (RMSE; Eq. 2) and mean absolute error (MAE; Eq. 3) for regression tasks. For regression tasks, the adjusted R^2^ is insensitive to insignificant independent variable which better capture the model performance^48^.

1 $$ R_{adjusted }^{2} = 1 - \frac{{\left( {1 - R^{2 } } \right)\left( {n - 1} \right)}}{n - k - 1} $$

2 $$ RMSE = \sqrt {\mathop \sum \limits_{i = 1}^{n} \frac{{\left( {\widehat{{y_{i } }} - y_{i} } \right)^{2} }}{n}} $$

3 $$ MAE = \frac{{\mathop \sum \nolimits_{i = 1}^{n} \left| {y_{i} - x_{i} } \right|}}{n} $$

For classification evaluation comparison, the confusion matrix, precision, recall and f1-score were also accounted for based on the ratio between true positive (TP), false positive (FP), true negative (TN), and false negative (FN). The precision is calculated based on the ratio between TP/TP + FP while the recall described the percentage between TP/TP + FN. The classification accuracy (TP + TN/TP + FN + TN + FP) and the f1-score (2*(precision * recall)/(precision + recall) are the most widely used metrics to evaluate the performance of machine learning algorithm for classification problem^23^.

## Results

### Petrophysical properties prediction

For simplicity purposes, all the algorithms involved in this study were implemented using default parameters which include only running the algorithm without specifying any related parameter. This is primarily to avoid the fine-tuning of hyperparameters associated with specific algorithms.

As a result, the first experiment used a linear regression-based algorithm to predict three different continuous logs: volume of shale (VSH), porosity (PHI), and mass percent of bitumen (W Tar). The first model was trained to predict the volume of shale (VSH), and it scored 71.15% adj_R^2^, 1.45% RMSE, and 8.32% MAE in the training phase. During the validation phase, the model received the following scores: 70.43% adj_R^2^, 1.46% RMSE, and 8.29% MAE (Table 1). The same model was then used to predict VSH on a completely separate dataset as a blind test of model performance. The model received the following scores: 71.93% adj_R^2^, 1.52% RMSE, and 8.73% MAE. This demonstrates very similar performance during training and generalization during the blind test (Table 1). In the porosity (PHI) prediction, the model predicted PHI with 70.29% adj_R^2^, 0.53% RMSE, and 3.13% MAE (Table 1). In the validation phase, the model achieved the following results: 69.68% adj_R^2^, 0.53% RMSE, and 3.13% MAE. In the blind test, the model achieved a slightly better performance where it achieved a 71.6% adj_R^2^, a 0.51% RMSE, and a 2.97% MAE (Table 1). The other continuous log to be predicted is the mass percentage of bitumen (W_Tar), which has sparse sampling within the available data set. As a result, predicting such a feature is expected to be more challenging due to insufficient overall data to train the model and evaluate the model’s performance. Using a similar Linear Regression algorithm to train the model, the following scores were reported during the training phase: adj_R^2^ is 12.96%, RMSE is 1.22%, and MAE is 3.43% (Table 1). When applied to the validation dataset, the model produced similar results: 13.55% adj_R^2^, 1.22% RMSE, and 3.43% MAE. The test results, on the other hand, revealed a dramatic drop in performance as follows: adj_R^2^ is 1.1%, RMSE is 1.22%, and MAE is 3.04% (Table 1). This result can be explained by lack of enough sampling for training and high bias within the available dataset. As a result, the model is unable to provide a reasonable prediction during the training, validation, and blind test phases.

**Table 1:** Summary of the performance of various supervised machine learning algorithms in regression tasks (VSH, PHI and W_Tar) for the blind test dataset. The best performing model is highlighted as bold values.

Target	Model	Metric	Score
VSH	Linear regression	Adjusted R^2^	71.93%
		RMSE	1.52%
		MAE	8.73%
	Gradient boosting machine	Adjusted R^2^	76.20%
		RMSE	1.40%
		MAE	8.09%
	AutoML_GBM	Adjusted R^2^	**78.77%**
		RMSE	**1.33%**
		MAE	**7.90%**
PHI	Linear regression	Adjusted R^2^	71.60%
		RMSE	0.51%
		MAE	2.97%
	Random forest	Adjusted R^2^	77.76%
		RMSE	0.45%
		MAE	**2.60%**
	AutoML_ Distributed random forest (DRF)	Adjusted R^2^	**80.45%**
		RMSE	**0.42%**
		MAE	**2.60%**
W_Tar	Linear Regression	Adjusted R^2^	1.10%
		RMSE	1.22%
		MAE	3.04%
	Gradient boosting machine	Adjusted R^2^	**67.85%**
		RMSE	**0.69%**
		MAE	0.53%
	AutoML_GBM	Adjusted R^2^	67.34%
		RMSE	0.71%
		MAE	**0.28%**

Significant values are in [bold].

**Figure 5:**
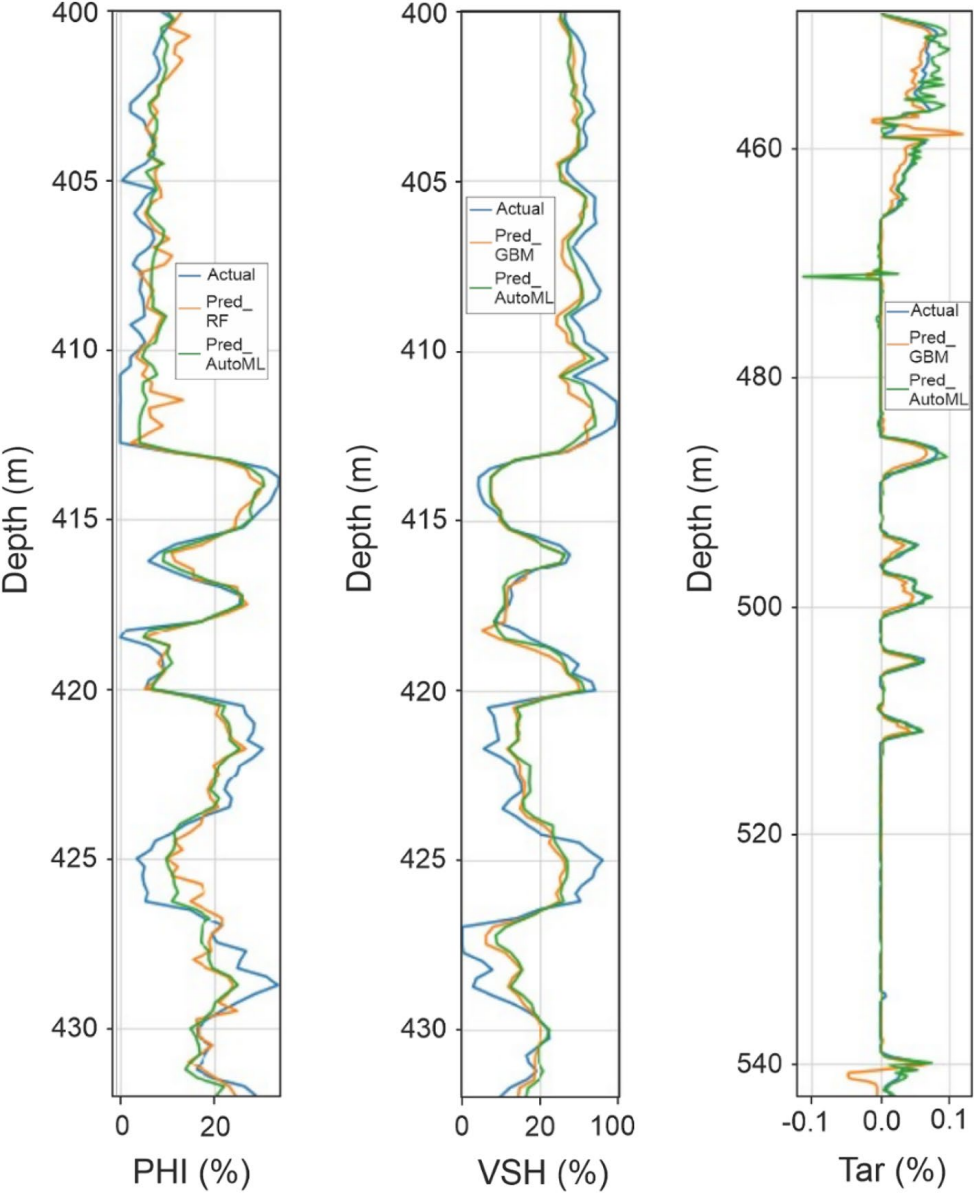
Plots showing the comparison between different ML algorithms and AutoML to the actual logs.

A similar approach has been used with various supervised machine learning techniques, but with more sophisticated and resource-intensive algorithms such as gradient boosting machine (GBM) and random forest (RF). Using the same training and validation datasets, these algorithms were employed to predict the three different parameters. Learning algorithms such as GBM and RF can be customized using a variety of hyperparameters, but for the sake of simplicity and avoiding hyperparameters, no pre-set parameters were used in this study. Instead, these learning models were applied only using the default set of parameters. The first feature (log) to be trained for, as in the previous workflow, is the volume of shale (VSH). The gradient boosting machine model performed better in this case than the Linear Regression (up to 5% improvement), scoring 76.2% adj_R^2^, 1.4% RMSE, and 8.09% MAE (Table 1). In this case, the random forest algorithm yielded higher scores for the other parameter, porosity (PHI), as follows: 77.76% adj_R^2^, 0.45% RMSE, and 2.60% MAE. The gradient boosting machine algorithm performed best in the volume of bitumen (W Tar) prediction, scoring 67.85% adj_R^2^, 0.69% RMSE, and 0.53% MAE despite the limited available data. This result shows a significant improvement from the simple linear regression model. It is therefore evident that the more advanced conventional machine learning models outperform the simple Linear Regression in all petrophysical properties prediction tasks evaluated in this study (Table 1). However, there are some discrepancies between the actual logs and predicted logs from RF and GBM as shown in Fig. 5. For example, the learning models underpredict porosity values especially in the high porosity interval and overpredict the values across the relatively tighter intervals.

Meanwhile, another parallel training model is constructed using the H_2_O tool to apply AutoML to the prediction of these three continuous logs. A similar approach is used by running the model with only simple default parameters (max_models = 10, max_runtime_sec = 400, seed = 1234) and using the same training, validation, and testing dataset for absolute performance comparison. The first feature to be explored is VSH, which is similar to the workflow used with supervised machine learning. In such a case, the AutoML approach tests a variety of supervised learning algorithms (ex. GBM, XGB, DRF) with various parameters. The primary model is then chosen using the best mean per class error metric. Similar to the conventional ML model for VSH, the GBM algorithm performed the best in this case and obtained the following results: 78.77% adj_R^2^, 1.33% RMSE and 7.90% MAE (Table 1). These metrics show an overall up 3% improvement when compared with conventional supervised machine learning with similar default parameters and show visually closer prediction when compared with the actual dataset (Fig. 5). The exact same approach using H_2_O tool was also applied to train the model to predict porosity. In this modelling, the AutoML process has identified distributed random forest (DRF) with (total number of trees = 50) as best fit given the run constraints. This allows a direct comparison with the conventional RF model for porosity (PHI) prediction. The DRF modelling achieved the following results in the blind test dataset: 80.45% adj_R^2^, 0.42% RMSE and 2.60% MAE (Table 1). This shows a similar magnitude of improvement (up to 3% improvement in R^2^) than the conventional RF model. Comparison with the actual test dataset reveals that the AutoML approach provides a much closer prediction than the conventional method (Fig. 5). The last continuous log to be modelled by AutoML is the W_Tar, in which the previous linear regression model exhibited poor correlation. The AutoML process has picked the GBM algorithm to be the fittest as per mean per class error score to predict the W_Tar similar to the conventional approach. The GBM model developed by the AutoML process has scored 67.34% adj_R^2^, 0.71% RMSE and 0.28% MAE despite the very limited training data available to train the model (Table 1) which shows a comparable performance with the conventional GBM model (Fig. 5).

### Lithofacies prediction

**Figure 6:**
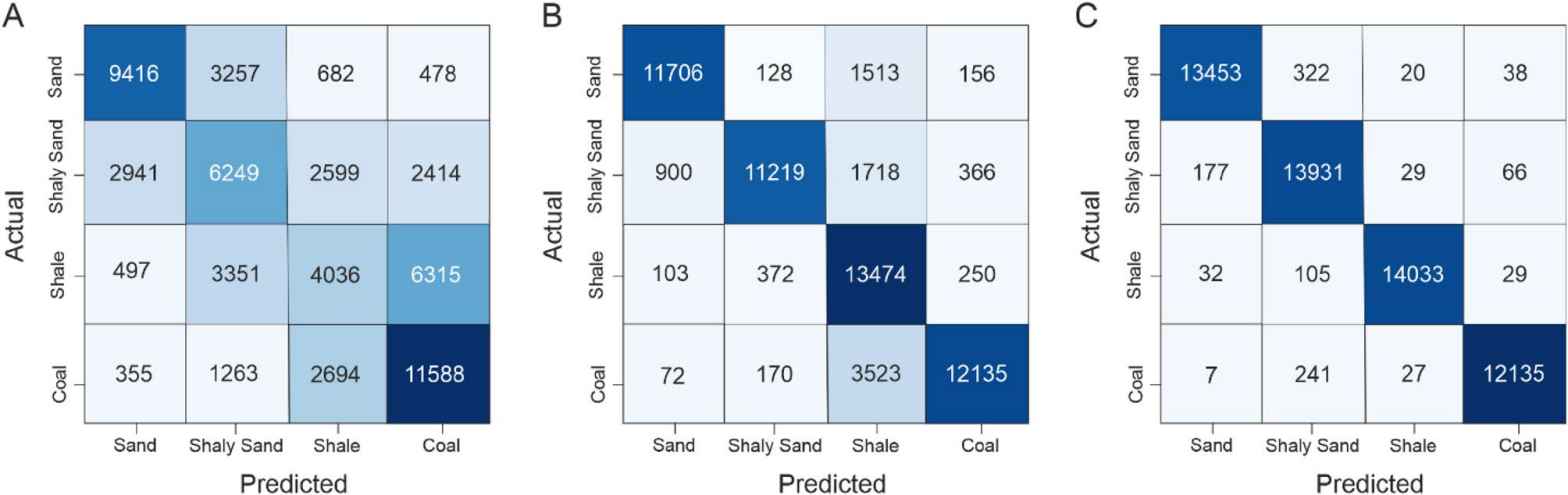
Confusion matrix of the three evaluated algorithms. (**a**) Logistic regression. (**b**) Gradient boosting Machine. (**c**) AutoML.

**Figure 7:**
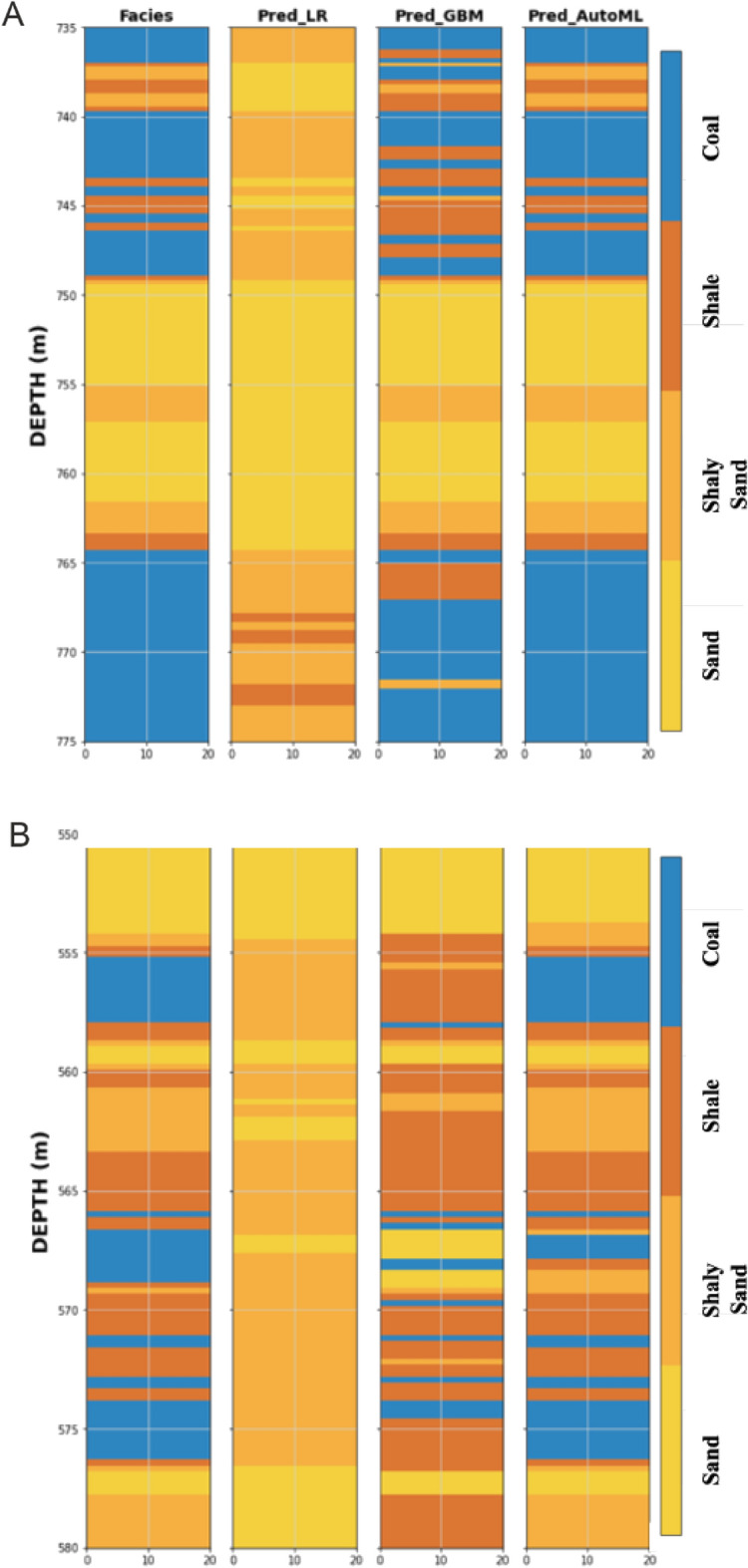
Comparison of lithofacies prediction using different machine learning algorithms in two different wells.

Similar to the previous workflow to generate model predicting the continuous logs, the next step is to construct models capable of predicting classification features (lithologies/facies). The predicted outputs from the first stage have been used as an input to predict lithology in addition to the previous set of logs used as training features. The first model created is supervised machine learning, which employs a simple logistic regression algorithm to avoid detailed hyperparameter tuning. The first model, which used logistic regression (LR), achieved a weighted average F1-score of 53% in testing phase (Table 2). In addition, it can be observed from the confusion matrix that the LR model achieved the highest precision (0.71) and recall (0.68) values are obtained with the sand class while the lowest precision (0.40) and recall (0.29) values were observed in the shale class (Table 2 and Fig. 6). Furthermore, the confusion matrix shows that the LR model struggles to properly evaluate three facies: sand, shaly sand, and shale. A comparison with the actual lithofacies data shows a poor correlation between the actual and predicted lithofacies from this LR model in the two blind tests well (Fig. 7A,B).

**Table 2:** Summary of facies prediction using different supervised machine learning algorithms and AutoML.

ML algorithm	Facies	Precision	Recall	F1-score
Logistic regression	Sand	0.71	0.68	0.7
	Shaly Sand	0.44	0.44	0.44
	Shale	0.4	0.29	0.33
	Coal	0.56	0.73	0.63
	Macro average	0.53	0.53	0.53
	Weighted average	0.53	0.54	0.53
Gradient boosting machine	Sand	0.92	0.85	0.88
	Shaly sand	0.94	0.79	0.86
	Shale	0.67	0.95	0.78
	Coal	0.92	0.76	0.83
	Macro average	0.86	0.84	0.84
	Weighted average	0.86	0.83	0.84
AutoML_GBM	Sand	0.98	0.97	0.98
	Shaly Sand	0.95	0.98	0.97
	Shale	0.99	0.99	0.99
	Coal	0.99	0.98	0.99
	Macro average	0.98	0.98	0.98
	Weighted average	0.98	0.98	0.98

On the other hand, the conventional gradient boosting machine (GBM) algorithm shows a significant improvement in the overall performance and achieved a weighted average of F1-score of 84% (Table 2). The model also achieved a relatively consistent high precision values across all the lithofacies (avg. 0.93) except shale (0.65) (Table 2 and Fig. 6). In contrast, among all the lithofacies, the shale facies achieved the highest recall value (0.95) while coal has the lowest recall value (0.76) (Table 2 and Fig. 6). From the confusion matrix of blind test dataset, it is evident that the GBM model has a particularly poor performance in predicting coal and misclassified it as shale (Figs. 6 and 7). Overall, the GBM model, even with default random hyperparameters, significantly improved prediction of all lithologies, particularly shale, which was poorly predicted using the conventional LR method. Another GBM model has been trained using H_2_O AutoML with the same input parameters. The AutoML-based model has shown a significant improvement when compared with the conventional GBM and achieved a weighted F1-score of 98% (Table 2). In addition, the AutoML approach provided a more consistent prediction across all the lithofacies, with high precision values ranging from 0.95 to 0.99 and the recall values ranging from 0.97 to 0.99 (Table 2 and Fig. 6). Furthermore, prediction results from the blind test wells and confusion matrix demonstrates that all various lithologies were properly assessed and correctly classified (Figs. 6 and 7).

## Discussion

Predicting various petrophysical properties such as the porosity and volume of shale as well as the categorical features such as lithofacies using AutoML has yielded promising potential as demonstrated in the study. The study shows that AutoML approach has outperformed conventional regression and advanced machine learning algorithms, such as RF and GBM, in the predictions of different petrophysical parameters (Figs. 5 and 7). Across all the predictions, the proposed AutoML has shown a significant improvement in lithofacies prediction (up to 15%) which is a very challenging task to predict, in particular when dealing with heterogeneous reservoirs^18,49^. In addition, the AutoML model can achieve such a high performance within a short period of time (less than 400 s) and minimal human intervention. A study by Palacios Salinas et al.^50^ further supports the advantage of AutoML in geosciences, specifically for remote sensing analysis. Furthermore, such an approach would allow to democratize advanced machine learning analysis in general and make it more accessible to non-machine learning experts which is geoscientist or petrophysicists in the case of subsurface well log interpretation.

**Figure 8:**
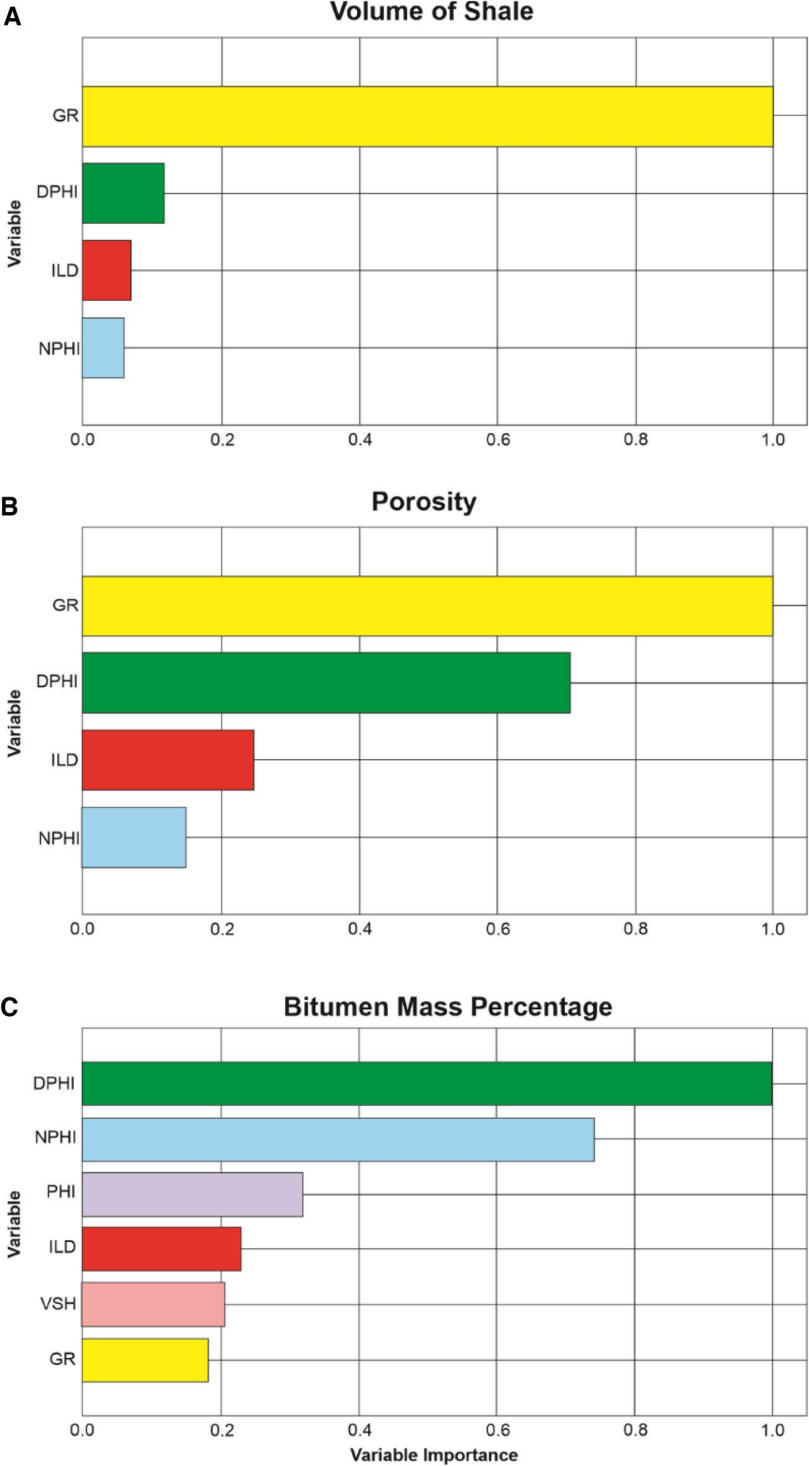
Histogram showing the feature importance ranking on the prediction of (**a**) volume of shale, (**b**) porosity and (**c**) bitumen mass percentage with AutoML.

**Figure 9:**
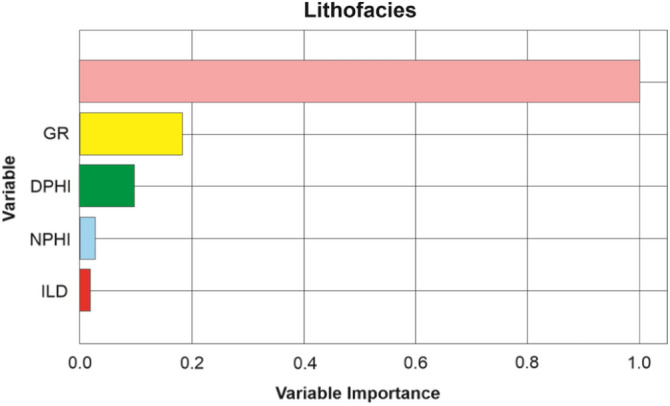
Histogram showing the feature importance ranking on the prediction of lithofacies.

Several major drawbacks of AutoML have been actively discussed in the literature, including high-cost training, overfitting, and low interpretability^26,27^. The high-cost training issue is mostly associated with the iterative training process, but with the current technology and advanced libraries, most AutoML could be trained in low-specification PC or personal laptop, as is the case for our study. The overfitting issue is commonly related to limited and unrepresentative dataset. In this study, we utilized close to five million data points collected from 2000 wells (Fig. 4) and the selection of validation and blind test dataset were curated carefully in order to have representative test sets. To address this, we extracted feature importance ranking from the best performed model to show how the model made the decision and prediction. This is a key information when building any learning model to better classify the relevant input logs and hence identifying relationship. Furthermore, it also provides a good insight into where some logs might actually be redundant and hence can be eliminated in the modelling workflow. For the VSH prediction, the gamma ray log was by far the most important log scoring around 74% which is not surprising since the volume of shale is typically driven by gamma ray calculations in conventional petrophysical analysis (Fig. 8a). The DPHI, ILD and NPHI logs scored 13%, 8% and 5%, respectively as the contributing factor in the calculation of VSH (Fig. 8a). This further supports that the AutoML model uses similar parameters that expert petrophysicists use to calculate VSH^51^. Similarly, both gamma ray and density logs play a significant role in predicting porosity with 48% and 34%, respectively (Fig. 8b). While density is commonly used to calculate total porosity from well logs, gamma ray is typically thought to have insignificant influence on the porosity calculation. In addition, neutron log has the lowest importance (18%) in the porosity prediction which is counterintuitive with the conventional petrophysical analysis (Fig. 8b). However, this phenomenon can be explained by the lithofacies types in this Athabasca oil sands field where the majority of lithofacies is sand, shale, and shaly sand in which the porosity can be significantly influenced by the gamma ray logs as illustrated in Fig. 4. Finally, according to the important feature report, both density and neutron porosity (DPHI & NPHI) logs play a major role in training the model to predict the W_Tar (Fig. 8c). For lithofacies prediction, the VSH emerges as the most influential parameters in the prediction of lithofacies. This is followed by the gamma ray and density logs. With the types of lithofacies analyzed in this study, it is understandable why the model place VSH as the most dominant feature than the GR in predicting lithofacies (Fig. 9). This information would be helpful for future studies that focus on well log interpretation in reservoir characterization.

## Conclusion

This study highlights the untapped potential of AutoML to accurately predict wireline logs and thus reservoir properties with a more robust and efficient workflow, and low carbon emission by eliminating time-consuming, manual analysis. Our findings show that the proposed AutoML method could predict different logs with high consistency and high levels of accuracy while using a legitimately simple workflow to implement. Overall, the AutoML processes are distinguished by the extreme simplicity they provide to novice users with limited experience in the fields of machine learning and data science. Another advantage is that it saves time and effort when experimenting with different algorithms and tuning their associated hyperparameters. The proposed model and library used in this study have the advantages from traditional machine learning because of their ability to can a large number of wells and different types of data and scalability for real-world deployments.

Furthermore, AutoML has provided useful insights into what specific algorithm could potentially be offered to solve a specific issue. The Gradient boosting algorithm, for example, is considered powerful in classification modeling, such as the facies/lithology prediction performed in this study. Furthermore, the feature importance percentage reporting embedded in the AutoML process is a useful tool for identifying relationships between various features (logs) and help to explain how the model base its decision to perform prediction. This will also result in better utilization of available data and improved data acquisition in future projects. Finally, this experiment shows that AutoML has a promising potential for improving formation evaluation using simple workflows. This can be validated by implementing AutoML workflow on more complex case studies in the future.

## Data Availability

All data used in this study is publicly available through https://ags.aer.ca/publication/spe-006. The source code can be made available upon reasonable request to the corresponding author.
