# Author Correction: Hierarchical automated machine learning (AutoML) for advanced unconventional reservoir characterization

**DOI:** 10.1038/s41598-023-46975-3

**Published:** 2023-11-09

**Authors:** Yousef Mubarak, Ardiansyah Koeshidayatullah

**Affiliations:** 1 Department of Geosciences, College of Petroleum Engineering and Geosciences, King Fahd University of Petroleum and Minerals, Dhahran, Saudi Arabia; 2 Saudi Aramco, 31311 Dhahran, Saudi Arabia; 3 Center for Integrative Petroleum Research, College of Petroleum Engineering and Geosciences, King Fahd University of Petroleum and Minerals, Dhahran, Saudi Arabia

Correction to: *Scientific Reports* 10.1038/s41598-023-40904-0, published online 24 August 2023

The original version of this Article omitted an affiliation for Yousef Mubarak. The correct affiliations are listed below:

Department of Geosciences, College of Petroleum Engineering and Geosciences, King Fahd University of Petroleum and Minerals, Dhahran, Saudi Arabia

Saudi Aramco, 31311, Dhahran, Saudi Arabia

Also there was an error in the acknowledgements section. The last digit of the Grant number was incorrect.

“The author acknowledged contribution from King Fahd University of Petroleum and Minerals, Saudi Arabia in providing the facilities to perform this study under a Research Grant from the SDAIA-KFUPM Joint Research Centre for Artificial Intelligence (CAI02564) awarded to A.K.”

now reads:

“The author acknowledged contribution from King Fahd University of Petroleum and Minerals, Saudi Arabia in providing the facilities to perform this study under a Research Grant from the SDAIA-KFUPM Joint Research Centre for Artificial Intelligence (JRCAI-RG-05) awarded to A.K.”

The original Article has been corrected.

